# Effect of a Broiler-Specific Light Spectrum on Growth Performance and Adrenocortical Activity in Chickens: A Pilot Study on a Commercial Farm

**DOI:** 10.3390/vetsci11120618

**Published:** 2024-12-02

**Authors:** Livio Galosi, Luca Todini, Laura Menchetti, Annaïs Carbajal, Rupert Palme, Nicola Ruggiero, Roberto Falconi, Alessandra Roncarati

**Affiliations:** 1 School of Biosciences and Veterinary Medicine, University of Camerino, Via Circonvallazione 93-95, 62024 Matelica, MC, Italy; livio.galosi@unicam.it (L.G.); luca.todini@unicam.it (L.T.); nicola.ruggiero@studenti.unicam.it (N.R.); roberto.falconi@unicam.it (R.F.); alessandra.roncarati@unicam.it (A.R.); 2 Department of Animal Health and Anatomy, Veterinary Faculty, Universitat Autònoma de Barcelona, Bellaterra, 08193 Barcelona, Spain; anais.carbajal@uab.cat; 3 Department of Biological Sciences and Pathobiology, University of Veterinary Medicine, Veterinärplatz 1, 1210 Vienna, Austria; rupert.palme@vetmeduni.ac.at

**Keywords:** chickens, broiler-specific light, LED, performances, corticosterone, dehydroepiandrosterone, animal-based measures

## Abstract

This study evaluated the effect of a broiler-specific light spectrum on productive performance corticosterone (fCC) and androgen dehydroepiandrosterone (fDHEA) concentrations in feathers, and glucocorticoid (GCMs) and androgen (AMs) metabolites in droppings of broilers. Two groups of female Ross 308 broilers were reared under white LED (WL, n = 9000) and broiler-specific LED (BSL, n = 9000) lights. The body weight (BW) of 150 randomly selected animals/groups was measured weekly. Droppings and feathers were collected at the end of the cycle (29 days) from 20 animals/group. The BSL group showed higher final BW than WL (1407 ± 11 vs. 1341 ± 15 g, respectively; *p* < 0.001) and higher indices of uniformity (76.8% vs. 61.2% animals in the 10% around the mean, respectively; *p* < 0.001). No difference between groups was found in fCC and fDHEA concentrations or in the fCC–fDHEA, indicating similar long-term HPA axis activity during the cycle. A higher concentration of GCMs was found in the BSL group, indicating higher glucocorticoid secretion before sampling, with neither a difference in AMs nor in GCMs–AMs. Finally, there was a positive correlation between fCC and fDHEA and between GCMs and AMs (*p* < 0.01). Our findings suggest that the use of broiler-specific light improved the productivity performances of chickens without long-term consequences on HPA activation. However, the results of this pilot study in a commercial farm setting must be interpreted with caution and need confirmation.

## Simple Summary

Light, among the environmental parameters, is a key factor for the well-being of broiler chickens in modern farming. It has the potential to influence a variety of physiological, immunological, and behavioural processes. In this study, we propose the use of a broiler-specific light spectrum, which is a combination of blue, green, and red LEDs, for chickens reared under commercial systems. We evaluated its effects on productivity performance and non-invasive indicators of hypothalamic–pituitary–adrenal (HPA) axis activity. Our findings suggest that the use of broiler-specific lighting significantly improves productivity performance, as indicated by the final mean body weight and uniformity index. While the analyses of droppings and feathers were feasible, further research involving replicates, sampling at multiple time points, and behavioural observations is needed to confirm their role as welfare indicators in farmed chicken.

## 1. Introduction

The use of coloured light in poultry breeding can have a marked impact on animal performances and growth, behaviour, and welfare, as reported by a wide literature review recently conducted by Soliman and El-Sabrout [1] and Abdel-Moneim [2]. Given that many studies produce contradictory results, it has become challenging to choose the best method of lighting to maximise broiler production. Some studies on broiler productivity suggest that light colour, as determined by wavelength, can affect broiler behaviour, with blue light having a calming effect, red light reducing feather pecking and cannibalism, and blue-green single or mixed LED light stimulating growth performance [3,4,5]. Thus, a lighting system that combines different colours could maximise the positive effects of every single colour.

The lighting system could have effects not only on production aspects but also on animal welfare. Animal welfare issues are raising growing awareness and concern among consumers and society in general, particularly with regard to intensive poultry farming [6]. Researchers and legislators are now focusing on methods for on-farm welfare assessment and, in particular, on animal-based measures (ABM) that can give indications of adaptive responses to different farming systems and management practices [7]. Among the physiological parameters, glucocorticoid hormones [8], mainly corticosterone in birds [9,10], are considered very valuable and have been used for decades to evaluate adaptive responses and animal welfare. They are the end product of the activation of the hypothalamic–pituitary–adrenal (HPA) axis, the main endocrine system involved in General Adaptation Syndrome [8,11,12]. Glucocorticoids (GCs) can be determined in different matrices. Blood concentrations acutely follow secretion by the adrenal gland, and in birds, a rise can be detected within a few minutes from the beginning of stressor exposure [13,14]. In chickens, Beuving and Vonder [15] described an increase 45 s after restraint. However, restraint is a stressor that can influence GC levels in the blood, creating a bias in evaluating the animal’s general well-being conditions [14,16,17,18]. Moreover, GCs secretion is affected by circadian rhythms [19] and can also increase in response to situations that do not negatively impact animal welfare [12,20,21]. HPA activation is, in fact, part of a natural and vital adaptive response to many stimuli, including physical activity, metabolic cues, parturition, courtship, copulation, and hunting [12,20,21]. Finally, in bird brains, intense steroid metabolism and neurosteroid production act on physiology and behaviour locally within the central nervous system [22] and can affect steroid concentrations in the blood [23]. Therefore, occasional measurements of blood corticosterone concentrations are not very meaningful for the assessment of mid- or long-term animal states of stress and welfare [24].

Measurement in faeces or droppings [25] is a non-invasive method that may dampen or resolve some biases inherent in the determination of GCs in blood. Depending on the gut passage time, circulating GCs are excreted in the faeces of mammals with a time lag of hours or days [26], 1–4 h in bird droppings [27]. The fluctuations in GC concentrations due to brief acute or episodic secretion of GCs can be thus smoothed out over longer time frames, and stress due to restraint is not detected [28]. The levels of GC metabolites in faeces are, therefore, a promising tool for obtaining cumulative information on the events experienced in the hours preceding sampling [28,29].

Corticosterone concentrations in feathers [30], instead, potentially provide a tool to evaluate long-term HPA activity, including in broilers [31]. Feather corticosterone (fCC) levels correspond to the cumulative hormone deposition related to circulating concentrations during feather growth, thus offering a time window of weeks [32]. Thus, they could be used to evaluate chronic stress, including that linked to the housing system.

In the continuous need to search multiple indicators for animal welfare assessment [33], and especially ABM, more recently, dehydroepiandrosterone (DHEA) and its sulfate (DHEAS) have attracted attention as potential biomarkers [34,35]. These androgen precursors are secreted by the zona reticularis of the adrenal glands in response to stressors and ACTH, at least in some species including humans [36], and display anti-GC biological effects [37].

Measuring GCs and DHEA(S) levels simultaneously, which exert many opposite biological effects, may be an important indicator of the net HPA activity. In humans, the cortisol–DHEA(S) ratio is considered an objective indicator of mental stress [38], while in farm animals, it is proposed as one potential biomarker of allostatic load and resilience [35,39]. While many studies are available in humans about DHEA(S) in both acute or prolonged stress [38], knowledge in non-human animals is rather scant [35] and lacking in poultry.

The hypothesis of the present study is that the lighting system can influence the performance of broiler chickens kept in intensive systems and that corticosterone and DHEA evaluated in feathers and GC and androgen metabolites in droppings can provide information on their adaptive responses. Therefore, the aim of the present study was to investigate the productive performance of chickens reared under a white or broiler-specific light system, consisting of a mix of blue, broad-spectrum green, and red LEDs, and obtain insights into medium and long-term activation of their HPA axis through the measurement of corticosterone and DHEA concentration in feathers and glucocorticoid and androgen metabolites in droppings.

## 2. Materials and Methods

### 2.1. Animals and Husbandry

The trial was conducted in two poultry houses (WL, white LED light; BSL, broiler-specific LED light), both with a floor area of 2000 m^2^ (length 112 × 18 m), located on a farm working in meat chicken production in the Marche Region, Italy. From a construction point of view, both buildings had the same equipment. The walls and the roof were made with 50 mm panels sandwiching internal polyurethane. The ventilation technology consisted of air-forced fans with a capacity of 45,000 m^3^/h installed at the ends of the walls. Tunnel type, with darkened windows with automatic opening, was adopted for the heating air generators, with three burners and ten radiant hoods. Cooling panels were also installed. Environmental parameters were monitored and recorded using a control unit (GT farm, Pola S.r.l., Soncino, CR, Italy) during the entire cycle. Before animal housing, the litter was prepared, covering the floor with a four cm deep layer of wood shavings in both sheds.

The study was conducted on a total of 18,000 Ross 308 female chickens (mean body weight (BW) ± standard error = 43 ± 0 g) after hatching (T0): one half was housed in the WL poultry house and the other half in the BSL poultry house. Females occupied half the area (1000 m^2^) of each shed (stocking density nine birds/m^2^), whereas the other half was reserved for males. The male chickens were not considered for the current trial because they were not of the same strain as the females.

WL and BSL animals were reared under the same environmental conditions and feeding plan. In particular, in both sheds, water was administered by five lines of nipples and feed was given ad libitum by four lines of feeders (7 cm/bird). Different diets were employed according to the phases of growth (Table 1). The feed conversion rate, communicated by the breeder, was similar and around 1.4:1 in both groups.

In both poultry houses, the microclimate parameters were maintained in ranges suitable to the phase of the chickens’ productive cycle (Table 2).

Throughout the cycle (29 days), the average weight, mortality, weight growth trend, and uniformity of the broilers in the two houses, which differ only in the lighting system, were evaluated at different times: T1 = 8 days; T2 = 15 days; T3 = 22 days; T4 = 29 days. In each poultry house, 150 female chickens were randomly selected and weighed at arrival and then weekly, using a manual poultry scale (BAT1, VEIT Electronics, Czech Republic).

### 2.2. Lighting

**Figure 1:**
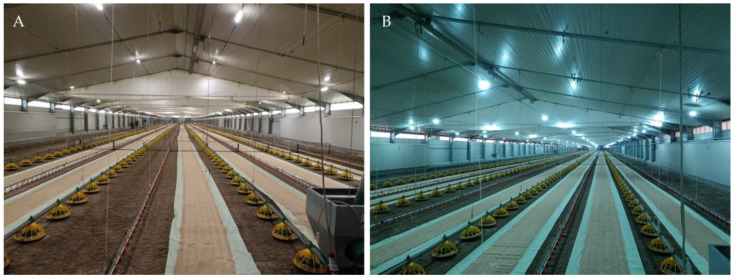
Poultry houses used in this trial. (**A**). Poultry house WL, equipped with compact fluorescent white light. (**B**). Poultry house BSL equipped with broiler-specific light (NatureDynamics System, ONCE by Signify, Philips).

In WL, lightning was ensured using a conventional system based on white LED. This system consisted of 84 lamps divided into four rows, with 21 lights spaced 6 m apart. The average illuminance was 80 lux. In BSL, the NatureDynamics System (ONCE by Signify, Philips, Plymouth, MN, USA) was applied, and 112 experimental LED lamps were installed, divided into four rows of 28 lights each. Each bulb in each row was 4.78 m apart from the next one. These lamps are based on a “Junglite recipe”, mixing three different LEDs: a blue, a broad-spectrum green, and a red LED. The lighting recipe combines the spectrum, schedule, and intensity of the three different LEDs that gradually change during the life of broilers. The average illuminance begins with bright light (50–80 lux) and then gets dimmed down (5–20 lux) as the broilers get older. The spectrum changes gradually during the life of the chickens, starting from a “Jungle Green” (including a spectrum with a white light that has a green hue to simulate a natural environment where young chicks are reared and where they should feel safe, and green and red spectra that would stimulate activity) to “Jungle Sky” (including a spectrum of a bluish-white light that is devoid of red light and designed to have a calming effect on chickens during the last stages of their cycle) [3,4,5]. Both poultry houses were lighted respecting the directive on the welfare of broilers in intensive breeding (Dir 2007/43/EC, implemented in Italy by DL 181/2010), ensuring a homogeneous distribution of the lighting. The lighting plan was the same in both poultry houses: 23 h of light and 1 h of darkness for the first 2 days, gradually increasing to 18 h of light and 6 h of darkness after 7 days, with 4 of these hours being continuous darkness (Figure 1).

### 2.3. Feather and Dropping Collection

At the end of the breeding cycle, feathers and droppings were sampled from 20 chickens randomly selected in each poultry house.

The primary sixth flight feather of the left wing (P6) was sampled by cutting close to the skin, avoiding any damage to the bird. This feather was chosen to obtain a uniform sample. It was fully grown (absence of blood in the calamus) before the first moult in all the animals. The adjacent P7 was moulted in many animals, while P5 was still growing and at different stages among the animals. Moreover, one P5 would not always be enough as a sample mass to be assayed. Each feather was washed under tap water and carefully dried with absorbent paper to remove surface contamination.

For dropping collection, each chicken was restrained in a paper box for a maximum of 15 min. Immediately after excretion, droppings were collected and refrigerated in a polystyrene box with ice. All samples were transported to the laboratory within two hours and frozen at −20 °C.

### 2.4. Steroid Assays

An optimised protocol for extracting corticosterone from feathers was used [40]. Each feather was measured (mm) to the nearest 0.1 mm (mean ± SD: 101.0 ± 3.9 mm) and weighed (mg) with a precision scale to the nearest 0.1 mg (mean ± SD: 72.3 ± 6.3 mg). After removing the calamus, feathers were mechanically minced, and the powder was incubated overnight in pure methanol. Samples were then centrifuged, and the supernatant evaporated. Dried extracts were reconstituted with EIA buffer solution provided by the enzyme immunoassay (EIA) kit (Corticosterone EIA kit; Neogen, Ayr, UK) and frozen at −20 °C until analysis. Two competitive EIA kits were performed to analyse corticosterone (Neogen^®^ Corporation, Ayr, UK) and DHEA (Arbor Assays^®^, Ann Arbor, MI, USA).

Droppings were extracted with a standard procedure utilizing 60% methanol [41]. GCMs and AMs were analysed with a cortisone and epiandrosterone enzyme immunoassay, previously described in detail [27,42]. Intraassay CVs in all EIAs were below 10%.

### 2.5. Statistics

Diagnostic graphics and Shapiro–Wilk and Levene’s tests were used to test assumptions and outliers. Extreme outliers (more extreme than Q1–3 * IQR or Q3 + 3 * IQR) were eliminated. In particular, one value was eliminated for body weight (BW), glucocorticoid metabolites (GCMs), androgen metabolites (AMs), and GCMs–AMs ratio. The BW and the fDHEA concentration were ln transformed, while the GCMs–AMs ratio in droppings was ln(x + 1) transformed to improve their distribution. Raw data are presented in the figures. Descriptive statistics were used to present the data as means and standard errors (SE) or median and ranges.

Changes in BW in the groups were analysed using Linear Mixed Models, including the subjects as a random factor. The model evaluated the effect of the group (two levels: WL and BSL), time (four levels: T1–T4), and their interaction, while the BW at T0 was included as a covariate.

The uniformity in BW (at each time) was evaluated using the Coefficient of Variation (CV = (standard deviation/mean) * 100), the Coefficient of Dispersion (COD = average absolute deviation as a percentage of the median), and the percentage of animals in the 10% and 15% range around mean [43]. The CVs of the two groups were compared using the method proposed by Forkman [44] and MedCalc Software [45]. The percentages of animals in the ranges around the mean were compared using Generalized Linear Model procedures using a binomial distribution and the logit link function. The chi-square procedure was used to analyse mortality rates.

The mean concentrations of dropping’ metabolites, fCC, and fDHEA were compared between groups using independent *t*-tests. A non-parametric approach (i.e., the Mann–Whitney test) was used to compare the droppings’ GCMs–AMs ratio (after ln transformation) and the fCC–fDHEA ratio.

Finally, the correlation between values was evaluated using Spearman’s coefficient (ρ). The strength of the correlation was interpreted as poor if ρ < │0.3│, medium if │0.3│ ≤ ρ < │0.5│, and large if ρ ≥ │0.5│ [46].

The statistical analyses were performed with SPSS 27.0 software (SPSS Inc. Chicago, IL, USA). A *p*-value < 0.05 was considered statistically significant.

## 3. Results

### 3.1. Body Weight and Productive Performances

**Figure 2:**
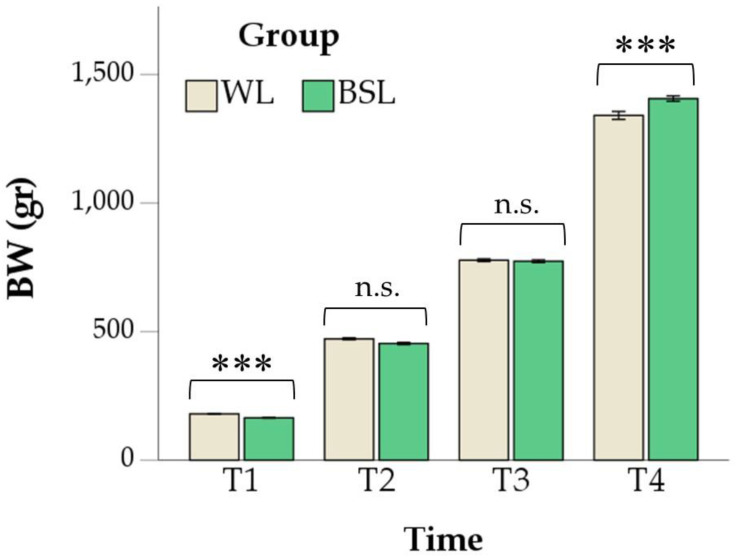
Body weight (BW) changes in the white LED (WL) and broiler-specific LED (BSL) groups. Values are means ± standard errors. The asterisks indicate significant differences between the two groups for each time point (*** *p* < 0.001; n.s. not significant). T1 = 8 days; T2 = 15 days; T3 = 22 days; T4 = 29 days.

The BW was influenced by time and its interaction with the group (*p* < 0.001). In particular, the WL group showed higher values at T1 (44 ± 0 vs. 42 ± 0 g, respectively; *p* < 0.001), while in the last time point, the BW was higher in the BSL group (1341 ± 15 vs. 1407 ± 11 g, respectively; *p* < 0.001; Figure 2).

Table 3 shows the indices calculated to evaluate the BW uniformity in the two groups. CV ranged from 7.88 to 14.00 in the WL group and from 7.99 to 11.96 in the BSL group, while COD ranged from 0.063 to 0.101 in the WL group and from 0.064 to 0.093 in the BSL group. In particular, at T4, the BSL group had a lower CV (*p* < 0.001) and COD than the WL group. At this time point, a greater percentage of the animals in the BSL group had a BW that fell close to the average (10% and 15% around the mean; *p* < 0.01). These findings indicate that BSL broilers were more uniform than WL broilers. Finally, the mortality rate was lower in BSL (0.97%) than in the WL group (2.14%; *p* < 0.001).

### 3.2. Corticosterone and DHEA in Feathers, Glucocorticoid and Androgen Metabolites in Droppings

**Figure 3:**
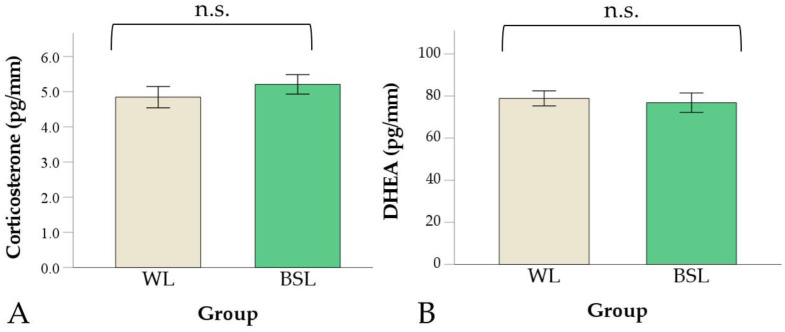
Corticosterone (**A**) and DHEA (**B**) in feathers in the white LED (WL) and broiler-specific LED (BSL) groups. Values are means ± standard errors (n.s. = not significant). DHEA = dehydroepiandrosterone.

**Figure 4:**
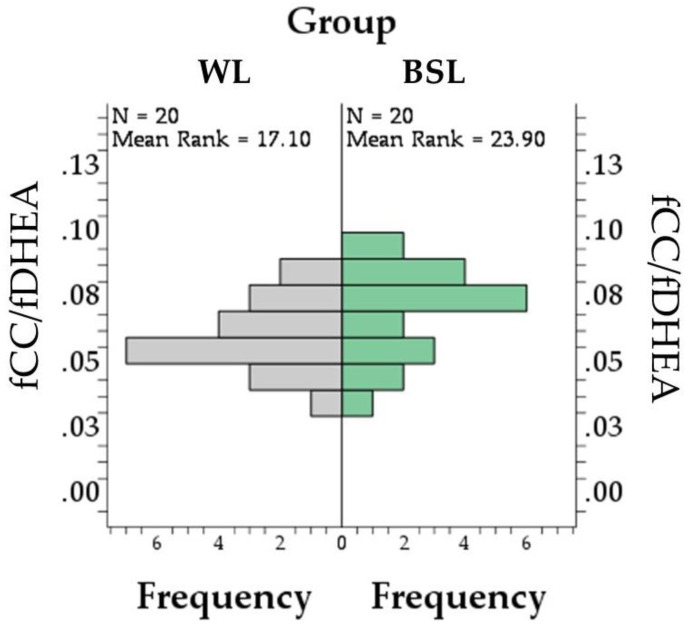
Pyramid chart and test statistics for the Mann–Whitney test comparing the fCC/fDHEA (expressed as pg/mm) distribution of white LED (WL) and broiler-specific LED (BSL) groups. Numbers on the x-axis indicate the frequency of the number of animals, while numbers on the Y-axis indicate the value of the fCC–fDHEA ratio. fCC = corticosterone concentrations in feathers; fDHEA = dehydroepiandrosterone concentrations in feathers.

A difference between groups was found for neither fCC concentrations (*p* = 0.690; Figure 3A), nor for fDHEA (*p* = 0.080; Figure 3B), nor for the fCC–fDHEA ratio (*p* = 0.068; Figure 4). The fCC–fDHEA ratio ranged from 0.04 to 0.08 in the WL group (median = 0.06) and from 0.4 to 0.09 in the BSL group (median = 0.07). Additional descriptive data are presented in Supplementary Table S1.

**Figure 5:**
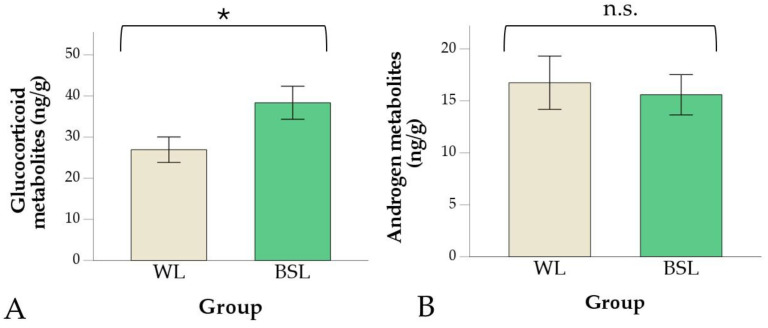
Glucocorticoid (**A**) and androgen (**B**) metabolites in droppings in the white LED (WL) and broiler-specific LED (BSL) groups. Values are means ± standard errors. The asterisk indicates significant differences between the two groups (* *p* < 0.05; n.s. = not significant).

**Figure 6:**
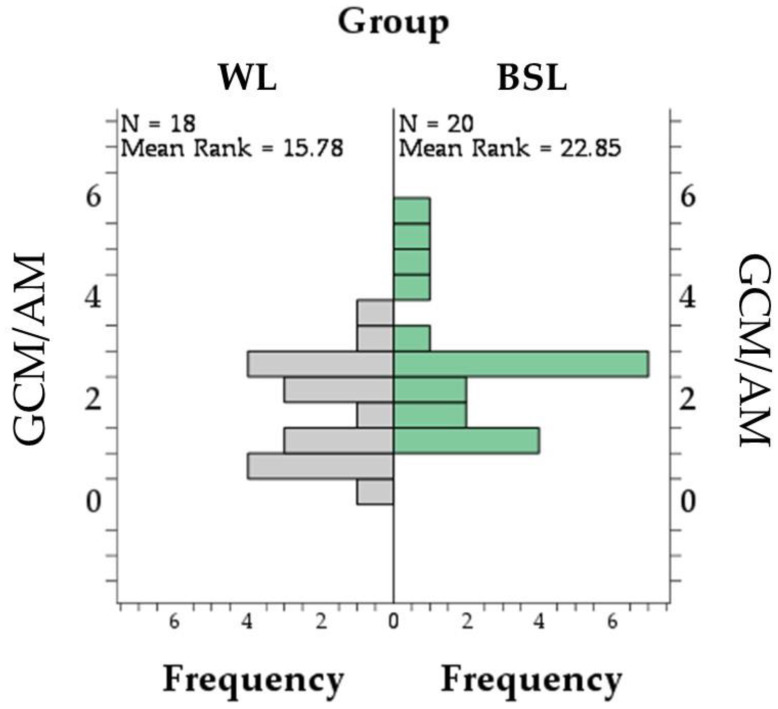
Pyramid chart and test statistics for the Mann–Whitney test comparing the distribution of glucocorticoid/androgen metabolites ratio (GCMs/AMs) in the droppings of white LED (WL) and broiler-specific LED (BSL) groups. Numbers on the x-axis indicate the frequency of the number of animals, while numbers on the Y-axis indicate the value of the ratio.

In droppings, the GCMs concentration was higher in the BSL group (38.4 ± 4.0 ng/g) than in the WL group (26.9 ± 3.1 ng/g; *p* = 0.031; Figure 5A), while no difference between groups was found in AM concentrations (*p* = 0.719; Figure 5B and Supplementary Table S1). Finally, the slight difference in the GCMs–AMs ratio (Figure 6 and Supplementary Table S1) did not reach statistical significance (*p* = 0.051), ranging from 0.3 to 3.95 in the WL group (median = 2.1) and from 1.4 to 5.8 in the BSL group (median = 2.7).

Regardless of the group, there was a positive, strong correlation between fCC and fDHEA (*p* < 0.01) and between GCMs and AMs (*p* < 0.01; Table 3). Conversely, GCM concentrations in droppings correlated moderately but negatively with fCC (expressed as pg/mg, ρ = −0.341) and fDHEA (expressed as pg/mg, ρ = −0.379, and pg/mm in Table 4) (*p* < 0.05).

## 4. Discussion

In poultry farming, lighting holds considerable importance for the advancement and modernisation of the industry, as it plays a significant role in the physiology and growth of broilers. Increasing attention has been paid to the impacts of lighting management on the growth performance, immune status, and welfare of broilers. For the first time, the present field study aimed to compare the productive performances and the medium- (glucocorticoid and androgens metabolites in dropping) and long-term (corticosterone and DHEA concentration in feathers) activation of the HPA axis of chickens. The trial, to increase its applicative nature, was carried out within a commercial farm in animals belonging to the same commercial strain (Ross 308), of the same weight, same sex (female), and of the same age, raised in shelters that differed by type of lighting only.

Regarding the final mean body weight and uniformity index, satisfactory productive results were obtained when the broiler chickens were reared in a poultry house equipped with broiler-specific LED light with respect to those exposed to conventional LED light. The maintenance of the same farming conditions and the feeding program suggests that the difference in light colour is of great importance to the growth of animals. This result agrees with most of the previous papers concerning this physical parameter [47]. Several studies reported a positive effect of green and blue light on BW, mainly evident at the early stage [5,47,48]. In the present trial, after the first week, broilers of the WL group were heavier than BSL ones, in agreement with Remonato Franco et al. [43], who used monochromatic green and/or blue light and observed heavier broilers under WL until 14 d of age. Conversely, the final BW (29 d) was higher in BSL than in WL broilers, according to most of the available papers regarding the use of monochromatic green or blue light [4]. Previous research reported that the body weight of broilers was higher under green light and blue light compared to WL from 5 to 21 days [49] and from 2 to 6 weeks [50], with higher weight gains at 2–5 weeks of age [47].

Another interesting result of our study was that animals exposed to the broiler-specific light system also showed an improvement in all indices of weight uniformity (i.e., lower CV and COD and a higher percentage of animals in the 10% and 15% range around the mean). It is necessary to underline that the current trial was performed on females, according to the chicken farming technique by separated sex used in Italy, whereas this technique is not customary abroad. The sex separation aims at obtaining a high uniformity of size at the end of the productive cycle, suitable for slaughtering to avoid off-range products. The broiler-specific LED light could contribute to maintaining the animals’ good distribution in the available area of the shed. This behaviour has been considered an ABM that can be used to evaluate the welfare state of chicks [51]. Less variability in final BW may also indicate less competition for feed access, considering that aggressive interactions at the feeder, although rare, can already start in broilers at two weeks of age [52]. However, our findings disagree with those of Remonato Franco et al. [43], who reported that light colour did not affect flock uniformity at 14 or 28 days of age. Further investigations, such as using behavioural observations, are necessary to confirm our hypothesis.

The positive effects of the broiler-specific light on the productive parameters were confirmed by the mortality, which was half compared to the WL group. Reducing mortality is an essential goal from both productive and ethical points of view. Firouzi et al. [53], in an experiment with various wavelengths ending at 42 d of age, reported the highest mortality rate for the blue light group (5.8%) and the lowest for the green light group (4.7%). Conversely, in most of the published papers, light wavelength did not affect the mortality of broilers [5,43,49,54,55].

Lighting requirements are always considered an important welfare standard that can give real benefits to poultry [7,51]. When investigating how to improve the lighting conditions in the poultry house to benefit growth performance and welfare status, the determination of hormones, such as corticosterone and DHEA, or their metabolites, sampled by a non-invasive method in feathers and droppings, can contribute to a better understanding of the animals’ capacities for adapting to poultry farming conditions.

Animal-based measures linked to productive aspects (i.e., BW, uniformity, and mortality) seem to suggest that the broiler-specific light system could improve animal welfare. Therefore, hormones and their metabolites indicating HPA axis activity were determined in the feathers and droppings, respectively. Concentrations of corticosterone and DHEA in feathers and their ratio were not different in the two experimental groups, indicating that throughout the weeks of feather growth, the cumulative hormone deposition, and thus adrenal steroid secretion, were on average similar between BSL and WL birds. It is not known when exactly the sampled feathers completed growth, and therefore, when hormone deposition from blood ceased. However, it likely occurs within the few days preceding the sampling. To our knowledge, DHEA(S) concentrations have never been investigated in poultry. DHEA in feathers has been previously assayed in red kites (*Milvus milvus*) for an ecotoxicology study performed by the same laboratory as the present work [40] and in Griffon vultures (*Gyps fulvus*), considering cortisol and DHEA measured by RIAs [56]. Blood DHEA(S) concentrations have been investigated in several wild avian species [23,57,58,59].

In droppings, the GCMs concentration was significantly higher in the BSL than in the WL birds, indicating that GCs secretion in the hours preceding the sampling was different between groups. Husbandry and handling of the two groups were similar. Therefore, we hypothesize that the zona fasciculata was differently activated in BSL and WL broilers coping with common environmental, husbandry, and/or social cues at the end of the cycle. Otherwise, an event not known to the authors occurred in the house where the animals of the BSL group were kept and activated the HPA axis. Regardless of the cause, metabolite levels in droppings reflect a limited time window and cannot represent welfare conditions throughout the entire livestock cycle. Despite the higher levels of GCMs in the droppings, all other ABMs (i.e., lower mortality, higher final BW, and uniformity) indicated a better state of well-being in the animals kept under the broiler-specific light system. Modern approaches to the evaluation of animal welfare, in fact, involve evaluations over time to establish whether the cumulative positive experiences exceed the cumulative negative ones (see the concept of *Quality of life* [60,61]). Further trials involving repeated sampling and continuous behaviour monitoring should be planned to investigate when GCs secretion and adaptive responses diverge in the long term.

Unlike GCM levels, no differences between groups were found in AM concentrations and the GCMs–AMs ratio in dropping. From the preliminary results of the present study, it seems that AMs could be a less sensitive indicator than GCMs for basal HPA axis activity in broiler chickens. Acute stress also did not result in plasma DHEA concentration changes in sparrows [62,63] and zebra finches [64]. Regarding AMs in droppings, the same assay as in the present work has been previously utilized to investigate the hypothalamus–pituitary–gonadal axis in male adult birds, such as Japanese quail [65,66,67], geese [68], and European stonechat [69]. Considering the age and sex of the birds in the present trial, the contribution of the gonads to circulating androgens should be negligible. Therefore, AMs in the droppings of our birds should be mainly of adrenal origin. In female geese, AM concentrations were related to gonadal activity as they increased after GnRH but not ACTH challenge [68].

Results of GCMs in droppings do not agree with previous literature data available for HPA axis activity evaluated from blood samples. Broilers kept under blue or green light showed lower plasma ACTH concentration in comparison with those under WL [50]. Ouyang et al. [70] reported that adult great tits (*Parus major*) exposed to WL at night had higher plasma corticosterone levels than individuals in green light or dark control. It has also been reported in male broilers that blue light [49] or both blue and green lights [54] promote growth by enhancing the synthesis of testosterone. However, in the present trial on females, neither differences in the concentration of AMs in droppings nor in that of DHEA in feathers were found. While there was no difference in fCC, significantly higher GCM concentrations and final BW were found in the BSL group. Previous studies in wild birds found negative correlations between fCC and BW in adults of Greenland barnacle geese (*Branta leucopsis*) [59] and between fCC vs. body size and body condition in nestlings of great tits (*Parus major*) [71]. On the other hand, plasma DHEA concentrations in two species of geese were neither correlated with BW [59] nor with body condition [72], while in song sparrow, baseline DHEA plasma levels were positively correlated with body condition during the breeding season [57].

Finally, regardless of the experimental groups, significant positive correlations were found between corticosterone and DHEA in feathers and GCM and AM levels in droppings. This may indicate a similar regulation of the two hormones’ secretion, both long-term and mid-term, confirming previous findings in wild sparrows, in which baseline corticosterone and DHEA in both jugular and brachial veins were positively correlated across all seasons [57]. Conversely, concentrations of GCMs in droppings correlated negatively with corticosterone and DHEA in feathers. It seems that individuals who had a relatively low HPA activity on average during the weeks of feather growth may have a relatively high GC secretion in the period (at least hours) preceding the sampling, and vice versa (i.e., individuals with relatively high fCC and fDHEA concentrations showed relatively low GCM concentrations).

The main limitation of this study was the use of a single house for each lighting system, as the lack of replicates reduces the reliability of the results. However, it was an applied study, on a commercial farm, which involved many animals and financial commitment for the farmer. This imposed restrictions and ethical and economic considerations. The large-scale effects of this innovative lighting system were completely unpredictable, and the implementation of a pilot study was necessary to avoid the risk of compromising the welfare of many animals and generating production losses for the farm. The results of this pilot study, therefore, pave the way for the use of an innovative lighting system but must be interpreted with caution and need confirmation. The research could also benefit from further evaluations. For example, precise monitoring of feather growth would be necessary to define the time window of the hormone deposition in the collected samples. The evaluations of metabolites in a single dropping sampling are not sufficient to define the farming conditions because they are only indicative of the situation during the previous hours. Thus, sampling at multiple time points could be planned. Finally, to comply with the multidimensional concept of animal welfare, behavioural assessments should accompany ABMs related to housing, productive performance, and physiological responses.

## 5. Conclusions

At the end of this trial, satisfactory productive results were obtained in terms of final mean body weight and uniformity index by rearing broiler chickens in a poultry house equipped with a broiler-specific light system including a mix of blue, broad-spectrum green, and red LEDs compared to those exposed to conventional white light. However, comparing studies of published papers is often difficult. Different animal strains, husbandry, and management conditions can affect different results in the various trials. Light, in particular, can impact animal physiology, welfare, and performances not only through various colours (wavelengths) but also through different photoperiodic programs, light intensities, and sources. Hormone assessments carried out in feathers could be used as ABM for welfare assessment as they provide insights into the cumulative activation of the HPA axis during the livestock cycle. However, in the present study, corticosterone and DHEA in feathers did not differ between groups and thus did not confirm the improvements in productive parameters obtained in chickens kept under green light. Replicates of the same protocol, longitudinal studies, and behavioural observations are needed to confirm these preliminary data and clarify relationships and changes in GC and DHEA(S) in various biological sample matrices, as well as the welfare over time.

## Figures and Tables

**Table 1:** Chemical composition (% as it is) and calculated metabolizable energy (ME) of the feeds administered to chickens in both poultry houses.

	Starter (1–8 d)	Grower I (9–15 d)	Grower II (16–22 d)	Finisher (23–29 d)
Protein	22.8	20	19.5	17.9
Lipids	5.5	6.6	8.2	7.6
Fibre	3.2	3.1	3.3	2.9
Ash	6.4	5.4	4.9	4.4
Lysine	1.48	1.29	1.26	1.13
Methionine	0.36	0.31	0.30	0.28
Calcium	0.94	0.77	0.66	0.59
Phosphorus	0.60	0.49	0.42	0.37
Sodium	0.16	0.16	0.16	0.15
ME (kcal/kg)	3010	3175	3180	3225

**Table 2:** Internal temperature, relative humidity, and air flow recorded during the trial in the two poultry houses equipped with white LED (WL) and broiler-specific LED (BSL).

Day	Temperature (°C)		Relative Humidity (%)		Air Flow (m^3^/h)	
	WL	BSL	WL	BSL	WL	BSL
Day 1	32.2	32.2	56	56	0.080	0.080
Day 8	29.5	29.4	54	55	0.420	0.420
Day 15	26.6	26.6	59	59	0.535	0.535
Day 21	23.1	23.2	63	64	0.652	0.652
Day 29	23.0	23.0	60	60	1.115	1.115

**Table 3:** Body weight uniformity indices in the white LED (WL) and broiler-specific LED (BSL) groups at different time points. Coefficient of variation (CV), coefficient of dispersion (COD), percentage of animals in the 10% and 15% around the mean. T1 = 8 days; T2 = 15 days; T3 = 22 days; T4 = 29 days.

Time	Index of Uniformity	Group		*p*-Value
		WL	BSL	
T1	CV (%)	10.40	8.30	0.006
	COD	0.086	0.065	-
	Animals in the 10% around the mean (%)	59.48	78.57	<0.001
	Animals in the 15% around the mean (%)	89.54	90.91	0.687
T2	CV (%)	9.82	11.96	0.017
	COD	0.080	0.093	-
	Animals in the 10% around the mean (%)	70.59	59.87	0.050
	Animals in the 15% around the mean (%)	87.58	80.26	0.084
T3	CV (%)	7.88	7.99	0.865
	COD	0.063	0.064	-
	Animals in the 10% around the mean (%)	79.61	81.05	0.752
	Animals in the 15% around the mean (%)	95.40	95.43	0.990
T4	CV (%)	14.00	9.20	<0.001
	COD	0.101	0.070	-
	Animals in the 10% around the mean (%)	61.18	76.82	0.004
	Animals in the 15% around the mean (%)	75.00	87.42	0.007

**Table 4:** Spearman’s ρ coefficient. fCC (corticosterone) and fDHEA (dehydroepiandrosterone) in feathers; GCMs (glucocorticoid) and AMs (androgen) metabolites in droppings.

	fCC (pg/mm)	fDHEA (pg/mm)	fCC/fDHEA ^1^	GCMs (ng/g)	AMs (ng/g)
**fDHEA (pg/mm)**	0.543 **	--			
**fCC/fDHEA ^1^**	0.514 **	−0.378 *	--		
**GCMs (ng/g)**	−0.294	−0.388 *	0.018	--	
**AMs (ng/g)**	−0.150	−0.247	0.002	0.502 **	--
**GCMs/AMs**	−0.059	−0.063	0.082	0.271	−0.597 **

**. Correlation is significant at the 0.01 level (2-tailed). *. Correlation is significant at the 0.05 level (2-tailed). ^1^. fCC and fDHEA calculated as pg/mm.

## Data Availability

The original contributions presented in the study are included in the article/Supplementary Material, further inquiries can be directed to the corresponding author/s.

## Supplementary Materials

The following supporting information can be downloaded at: https://www.mdpi.com/article/10.3390/vetsci11120618/s1, Table S1. Descriptive statistics for the parameters relating to the sampling of feathers and droppings in the white LED (WL) and broiler-specific LED (BSL) groups.
