# Supplementary material for: Effect of a Broiler-Specific Light Spectrum on Growth Performance and Adrenocortical Activity in Chickens: A Pilot Study on a Commercial Farm
Source: Vet Sci. 2024 Dec 2;11(12):618. doi: 10.3390/vetsci11120618 (PMC11680236; doi:10.3390/vetsci11120618)
Supplement: Supplementary file 1 [file vetsci-11-00618-s001.zip › vetsci-3222514-supplementary.pdf]

Effect of a Broiler-specific light spectrum on Growth Performance and Adrenocortical Activity in Chickens: a pilot study on a commercial farm

Livio Galosi, Luca Todini, Laura Menchetti, Annaïs Carbajal, Rupert Palme, Nicola Ruggiero, Roberto Falconi, Alessandra Roncarati

Supplementary Material

Table S1. Descriptive statistics for the parameters relating to the sampling of feathers and droppings in the white LED (WL) and broiler-specific LED (BSL) groups. DHEA =dehydroepiandrosterone.

Matrix	Parameter	Group			
		WL		BSL	
		Mean	Standard Error of Mean	Mean	Standard Error of Mean
Feathers	Length of feathers (mm)	100	1	102	1
	Weight of feathers (mg)	60.5	2.1	67.6	1.5
	Corticosterone concentration (pg/mg)	8.14	0.54	7.86	0.41
	Corticosterone concentration (pg/mm)	4.85	0.30	5.21	0.28
	DHEA concentration (pg/mg)	133.37	7.42	116.60	7.27
	DHEA concentration (pg/mm)	78.89	3.57	76.83	4.64
	Corticosterone/DHEA (pg/mg)	0.06	0.00	0.07	0.00
	Corticosterone/DHEA (pg/mm)	0.06	0.00	0.07	0.00
Droppings	Glucocorticoid metabolites (ng/g)	26.93	3.10	38.36	4.01
	Androgen metabolites (ng/g)	16.74	2.56	15.59	1.94
	Glucocorticoid/Androgen metabolites	1.92	0.24	2.84	0.29
